# Denatured M13 Bacteriophage‐Templated Perovskite Solar Cells Exhibiting High Efficiency

**DOI:** 10.1002/advs.202000782

**Published:** 2020-08-05

**Authors:** Hao‐Sheng Lin, Jong‐Min Lee, Jiye Han, Changsoo Lee, Seungju Seo, Shaun Tan, Hyuck Mo Lee, Eun Jung Choi, Michael S. Strano, Yang Yang, Shigeo Maruyama, Il Jeon, Yutaka Matsuo, Jin‐Woo Oh

**Affiliations:** ^1^ Department of Mechanical Engineering School of Engineering The University of Tokyo Tokyo 113‐8656 Japan; ^2^ Department of Chemical Engineering Massachusetts Insititute of Techonology Cambridge MA 02139 USA; ^3^ Research Center for Energy Convergence and Technology Pusan National University Busan 46241 Republic of Korea; ^4^ Department of Nano Fusion Technology Pusan National University Busan 46241 Republic of Korea; ^5^ Department of Materials Science and Engineering KAIST 291 Daehak‐ro, Yuseong‐gu Daejeon 34141 Republic of Korea; ^6^ Department of Materials Science and Engineering and California Nano Systems Institute University of California Los Angeles CA 90095 USA; ^7^ Research Center for BIT Fusion Technology Pusan National University Busan 46241 Republic of Korea; ^8^ Energy NanoEngineering Laboratory National Institute of Advanced Industrial Science and Technology (AIST) Tsukuba 305‐8564 Japan; ^9^ Department of Chemistry Education Graduate School of Chemical Materials Institute for Plastic Information and Energy Materials Pusan National University 63‐2 Busandaehak‐ro Busan 46241 Republic of Korea; ^10^ Institutes of Innovation for Future Society Nagoya University Furo‐cho, Chikusa‐ku Nagoya 464‐8603 Japan

**Keywords:** bioelectronics, crystal growth templates, M13 bacteriophages, perovskite solar cells, wild viruses

## Abstract

The M13 bacteriophage, a nature‐inspired environmentally friendly biomaterial, is used as a perovskite crystal growth template and a grain boundary passivator in perovskite solar cells. The amino groups and carboxyl groups of amino acids on the M13 bacteriophage surface function as Lewis bases, interacting with the perovskite materials. The M13 bacteriophage‐added perovskite films show a larger grain size and reduced trap‐sites compared with the reference perovskite films. In addition, the existence of the M13 bacteriophage induces light scattering effect, which enhances the light absorption particularly in the long‐wavelength region around 825 nm. Both the passivation effect of the M13 bacteriophage coordinating to the perovskite defect sites and the light scattering effect intensify when the M13 virus‐added perovskite precursor solution is heated at 90 °C prior to the film formation. Heating the solution denatures the M13 bacteriophage by breaking their inter‐ and intra‐molecular bondings. The denatured M13 bacteriophage‐added perovskite solar cells exhibit an efficiency of 20.1% while the reference devices give an efficiency of 17.8%. The great improvement in efficiency comes from all of the three photovoltaic parameters, namely short‐circuit current, open‐circuit voltage, and fill factor, which correspond to the perovskite grain size, trap‐site passivation, and charge transport, respectively.

## Graphical abstract

The M13 bacteriophage functions as an effective perovskite growth template and a passivator in perovskite solar cells. This is owing to its filamentous and uniform dimension, as well as the amino acids on its surface. These effects enhance when the M13 viruses are denatured at high temperature. The efficiency increases from 17.8% to 20.1% upon addition of the denatured viruses.

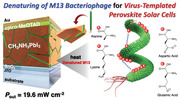

Nature‐inspired biomaterials and their device application are a promising field of research in the light of the raw material shortage and environmental problems of today.^[^ ^1^, ^2^ ^]^ The M13 bacteriophage, which is the single‐stranded DNA viruses, is a biomaterial that has recently shown great potential in electronics application.^[^ ^3^, ^4^, ^5^, ^6^, ^7^, ^8^, ^9^, ^10^ ^]^ The M13 bacteriophage has a structure of single‐stranded DNA enclosed in a cylindrical capsid with 2700 copies of the pVIII protein on its body and five copies of the pIII protein on one end. The pVIII proteins are oriented in a way that the surface of the M13 bacteriophage has both peptide termini, i.e., amino ends and carboxyl ends. For this reason, the M13 bacteriophage has demonstrated metal template capability using the arms along the shaft of their body to bind to charged substances.^[^ ^11^, ^12^, ^13^, ^14^, ^15^, ^16^, ^17^, ^18^ ^]^ The flexible and filamentous nature of the M13 bacteriophage with the high aspect ratio of a diameter of ≈6.5 nm to a length of ≈880 nm indicates that they are excellent candidates for a crystal growth template.^[^ ^3^, ^4^ ^]^

In the wake of the sustainable society, perovskite solar cells (PSCs) have emerged as the next‐generation energy source.^[^ ^19^ ^]^ The high power conversion efficiency (PCE) of PSCs originates from the high absorption coefficient,^[^ ^20^ ^]^ long‐range diffusion length,^[^ ^21^ ^]^ and high defect tolerance^[^ ^22^ ^]^ of the lead‐halide perovskite photoactive materials. To improve the PCE further, the perovskite crystal size should be enlarged and their grain boundaries must be passivated.^[^ ^23^, ^24^, ^25^ ^]^ Numerous researchers around the world have reported use of polymers^[^ ^26^, ^27^, ^28^ ^]^ and carbon nanotubes^[^ ^29^, ^30^ ^]^ as the perovskite crystal growth templates and cross‐linkers to achieve these.

In this work, we demonstrate the use of the M13 bacteriophage as the perovskite crystal growth template. Unlike polymers and carbon nanotubes, the M13 bacteriophage has a uniform length of ≈880 nm, which can surround the perovskite grains with a diameter of 300 nm to 1 µm effectively. This is a crucial advantage as not only the size but also the homogeneity of the perovskite grain is considered important for the perovskite film quality.^[^ ^26^, ^27^, ^28^, ^29^, ^30^ ^]^ The polymeric growth templates possess nonuniform side chains and their length exceeds the perovskite grain circumferences that aggregations and ineffective placement among the grains can undermine the perovskite film quality.^[^ ^31^, ^32^ ^]^ Carbon nanotubes are better in this regard as they are shorter in length and rarely aggregate owing to the surrounding surfactants.^[^ ^33^, ^34^ ^]^ Still, carbon nanotubes have nonuniform tube lengths and metallic impurities, the latter of which results in charge recombination of electrons and holes as the metallic carbon nanotubes have small bandgaps.^[^ ^35^, ^36^ ^]^ The M13 bacteriophage, however, does not have problems. Moreover, the synthesis of the M13 bacteriophage is chlorinated organic solvent‐free and significantly low‐cost compared with the polymers and carbon nanotube additives. Such advantages make the M13 bacteriophage an excellent choice of additives for the organohalide metal perovskite films.

Herein, the wild‐type M13 bacteriophage without any genetic engineering was mixed in the perovskite solution prior to spin‐coating. The carboxylic and the amino groups of the four types of amino acids on the surface of M13 bacteriophage formed Lewis base coordinations to Pb^2+^. This is not surprising because amino acid in polypeptides^[^ ^37^, ^38^, ^39^, ^40^, ^41^ ^]^ are known to passivate perovskite grain boundaries and promote a favorable crystal orientation for charge transport. The effective coordination to Pb^2+^, and the uniform and apposite dimension of the M13 bacteriophage, the single‐stranded deoxyribonucleic acid (DNA) virus enabled the formation of homogeneous and large crystal grains. As the M13 bacteriophage in room temperature (r.t.) exists in tertiary and quaternary structures, they were straightened by breaking the inter‐and intramolecular interactions using heat energy to maximize the crystal growth template effect. While the reference PSCs gave a PCE of 17.8%, the M13 virus‐added PSCs exhibited a PCE of 18.7%. When the M13 virus‐added perovskite precursor solution was heated at 65 °C before spin‐coating, the PCE increased to 19.7%. The efficiency further improved to 20.1% upon heating the virus‐added perovskite precursor solution at 90 °C. Such high efficiency resulted from a short‐circuit current density (*J*_SC_) of 24.7 mA cm^−2^, an open‐circuit voltage (*V*_OC_) of 1.09 V, and a fill factor (FF) of 0.75. The high *J*_SC_ came from the increased incident photon‐to‐current efficiency attributed to the large crystal grains as well as the light scattering effect of the viruses.^[^ ^42^ ^]^ The high *V*_OC_ came from the passivation of the grain boundaries between perovskite crystals, which reduced the shallow trap sites and nonradiative recombination.^[^ ^43^, ^44^, ^45^, ^46^, ^47^, ^48^, ^49^, ^50^, ^51^ ^]^ The high FF came from the improved charge transport ascribed to both the favorable orientation of the perovskite crystals and the passivation effect of the crystal grains by the M13 bacteriophage.^[^ ^52^, ^53^ ^]^ Within the best of our knowledge, this is the first bacteriophage application to PSCs and the first biomaterial to be used as the perovskite growth template and the passivator. This finding will widen the material selection for the perovskite crystal growth template and the passivator. We are confident that this work will serve as the milestone for the bioelectronics application, especially in energy device application.

The wild‐type M13 bacteriophage was cultured by a mass‐amplification protocol in our laboratory.^[^ ^54^ ^]^ The amplified M13 bacteriophage was purified by centrifugation, followed by dispersion in anhydrous dimethylsulfoxide (DMSO) (Figure S1, Supporting Information). Normal‐type PSCs in a structural configuration of indium tin oxide (ITO)/SnO_2_/CH_3_NH_3_PbI_3_/2,2′,7,7′‐tetrakis(*N,N*‐di‐*p*‐methoxyphenylamine)‐9,9′‐spirobi‐fluorene (spiro‐MeOTAD)/Au were fabricated. The wild‐type M13 bacteriophage was mixed directly into the solution of CH_3_NH_3_PbI_3_ (MAPbI_3_) (**Figure** **1**a).^[^ ^13^, ^55^, ^56^ ^]^ A small amount (5.9 wt%) of DMSO is typically added to the MAPbI_3_ solution, in which *N*,*N*‐dimethylformamide (DMF) is the solvent, to comply with the optimal molar ratio of 1:1:1mol% (MAI, PbI_2_, and DMSO).^[^ ^57^ ^]^ Therefore, the M13 bacteriophage was added to DMSO in different concentration, which was followed by addition to MAPbI_3_ in DMF to maintain the molar concentration of DMSO (5.9 wt%) (Table S1, Supporting Information). The 0.02 wt% concentration of M13 bacteriophage in DMSO gave the highest PCE when applied to PSCs (Table S2, Supporting Information), which was 18.7%, with a *J*_SC_ of 24.4 mA cm^−2^, a *V*_OC_ of 1.06 V, and a FF of 0.72 (**Table** **1**: entry 1; Figure S2, Supporting Information). During the investigation, we found that filtering the virus solution enhanced the PCE, which meant that there were virus aggregations present in the virus solution and the removal of those aggregates improved the device performance (Table S2, Supporting Information). This implied that unentangling the virus aggregates could improve the device performance. This is because the amino acids on M13 bacteriophage are known to form a quaternary structure due to the hydrogen bonding and polar interactions with other bacteriophages and within themselves (Figure 1a). Applying heat has been known as one of the accepted methods of breaking those bonds, also known as denaturing. Heating the virus‐added MAPbI_3_ solution at certain temperatures prior to spin‐coating improved the PCE greatly (Figure 1b,c; and Table 1: entry 3 and 4). When the virus‐added MAPbI_3_ solution was heated at 65 °C for 15 min, PSCs gave an improved PCE of 19.7% with a *J*_SC_ of 24.6 mA cm^−2^, a *V*_OC_ of 1.07 V, and an FF of 0.74 (Table 1: entry 3). Raising the temperature to 90 °C improved the PCE even further to 20.1% with a *J*_SC_ of 24.7 mA cm^−2^, a *V*_OC_ of 1.09 V, and a FF of 0.75 (Table 1: entry 4). The champion device exhibited the stabilized power output of 19.6% obtained from the maximum power tracking data (Figure 1d). From the statistical analysis, it is clear that all three photovoltaic parameters contributed to the PCE enhancement (Figure S3, Supporting Information). Raising the temperature above 90 °C led to the evaporation of the solvents as well as the total denaturing of the M13 bacteriophage, so 90 °C was the most optimal temperature.^[^ ^58^, ^59^ ^]^ Moreover, it is worth noting that the hysteresis of the virus‐added PSCs was lower than that of the reference devices and the hysteresis index decreased with the increase in the temperature (Figure S4, Supporting Information). Though the improvement is marginal, this may indicate enhanced passivation effect arising from reduced trapped charge.^[^ ^60^, ^61^ ^]^ It should be mentioned that the heated perovskite solution without the virus resulted in the similar photovoltaic performance as the reference devices (Table 1: entry 5).^[^ ^59^, ^62^, ^63^ ^]^ In the following paragraphs, we discuss the mechanism behind the enhancement of the photovoltaic parameters upon addition of the M13 bacteriophage and the heat treatment.

**Figure 1:**
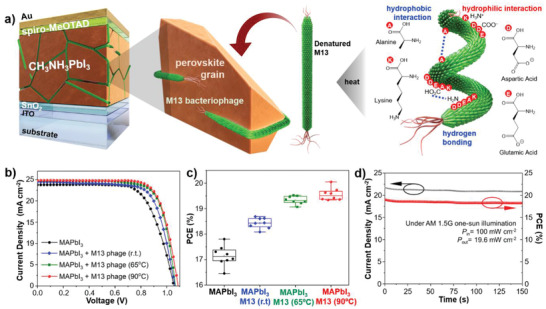
a) Illustration of normal‐type PSCs structure used in this work with the denatured M13 bacteriophage as the perovskite growth template. b) Reverse scan *J*–*V* curves of the best‐performing PSCs using the M13 bacteriophage‐added solution heated in different temperature. c) The box plot chart the PCE statistical analysis of the PSCs with the M13 bacteriophage under different heat treatment. d) Stabilized current density and maximum power point tracking of the 90 °C heated M13 bacteriophage added PSCs measured under an applied voltage of 0.88 V and AM 1.5G 1 sun illumination (100 mW cm^−2^).

**Table 1:** Photovoltaic parameters of the PSCs using 0.02 wt% of M13 bacteriophage added perovskite solution heated at different temperatures under 1 sun (AM 1.5 G, 100 mW cm^−2^). Average values with standard deviation were obtained from the devices fabricated in the same batch (36 devices). The statistical analysis of PCE is provided in Figure 1b, other photovoltaic parameters are shown in Figure S3 (Supporting Information)

Entry	Perovskite	*J*_SC_ [mA cm^−2^]	*V*_OC_ [V]	FF	PCE_best_	PCE_average_
1	Ref.	23.6 ± 0.1	1.035 ± 0.008	0.700 ± 0.009	17.8%	17.1 ± 0.4%
2	M13 phage (r.t.)	24.4 ± 0.2	1.058 ± 0.004	0.706 ± 0.009	18.7%	18.4 ± 0.2%
3	M13 phage (65 °C)	24.6 ± 0.2	1.076 ± 0.005	0.733 ± 0.006	19.7%	19.4 ± 0.2%
4	M13 phage (90 °C)	24.7 ± 0.1	1.083 ± 0.006	0.737 ± 0.007	20.1%	19.7 ± 0.2%
5	Ref. (90 °C)	23.2 ± 0.3	1.031 ± 0.010	0.700 ± 0.010	17.4%	16.8 ± 0.6%

The effect of heat on the M13 bacteriophage was studied using scanning electron microscope (SEM) to observe a virus film, which was dried at r.t. and 90 °C each. It was observed that drying the viruses at r.t. led to an aggregated film (**Figure** **2**a). On the other hand, drying the viruses at 90 °C led to a porous film (Figure 2b). This means that the heated viruses are physically stretched out. This is more clearly observed in the dynamic light scattering (DLS) measurement. The intensity distribution of DLS shows that the M13 bacteriophage in DMF at r.t. displays typical multiple peaks of the virus, which comes from the various quaternary structures and aggregations,^[^ ^64^ ^]^ whereas the M13 bacteriophage heated at 75 °C (75 °C was the highest temperature the DLS machine could reach) show a much sharper peak indicating a particle size of around 100 nm (Figure 2c). The difference in size and distribution can be more clearly seen in the number distribution plot. The heated M13 bacteriophage exhibits a smaller particle size and narrower distribution than the M13 bacteriophage in r.t. (Figure 2d). This proves the denaturing of the viruses upon heat treatment.

**Figure 2:**
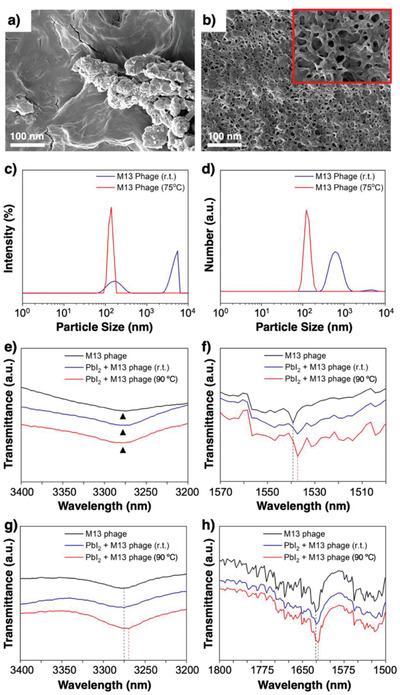
SEM images of the dried M13 bacteriophage a) without heat treatment and b) with heat treatment at 90 °C. Magnified image is provided in the inset. c) DLS intensity distribution and d) number distribution of the M13 bacteriophage in r.t. (red) and with the heat (blue) treatment. FTIR spectra of e) *ν*_(N—H)_ and f) *ν*_(C=O)_ for the M13 bacteriophage (black), the adduct powder of PbI_2_ mixed with the M13 bacteriophage (blue) and the heat‐treated adduct powder (red) in solid state. FTIR spectra of g) *ν*_(N—H)_ and h) *ν*_(C=O)_ the M13 bacteriophage (black), the adduct powder of PbI_2_ mixed with the M13 bacteriophage (blue) and the heat‐treated adduct powder (red) in solution state.

The interaction between the M13 bacteriophage and PbI_2_ was assessed using the Fourier‐transform infrared (FTIR) spectroscopy. The wild‐type M13 bacteriophage has been reported to possess a negatively charged N‐terminal domain, an intermediate domain, and a passively charged domain.^[^ ^4^ ^]^ Out of these three domains, only the negatively charged N‐terminus is exposed to media.^[^ ^65^ ^]^ The interaction between Pb^2+^ and the M13 bacteriophage comes from the electron lone pair on the amino groups of N‐terminal alanine (A) and lysine (K) and the negative charge on the carboxylic acid groups in aspartic acid (D) and glutamic acid (E) coordinating to Pb^2+^ (Figure 1a). The adduct powder of PbI_2_ mixed with the M13 bacteriophage shows a negligible shift of the N—H stretch at ≈3270 cm^−1^ (Figure 2e). However, the downshift is clear for the C=O stretch at ≈1538 cm^−1^, though heating the powder did not change the shift (Figure 2f). The fact that the C=O stretch shift was more visible than the N—H stretch shift implies that the interaction between the carboxylic groups and PbI_2_ is more dominant than that between the amino groups and PbI_2_. The M13 bacteriophage contains 4 different amino acids, A, K, D, and E all in different proportions. We conjecture that that K induces strong interaction of the amino group while D and E are likely to induce strong interaction of the carboxyl end. Hence the interaction intensity of N and O appear different on FTIR. When the M13 bacteriophage was dissolved in DMF, the interaction with PbI_2_ was more visible. The N—H stretch shifted greatly upon heating (Figure 2g; and Figure S5a, Supporting Information). The C=O stretch showed a downshift as well, but the shift was not as noticeable as that of the N—H (Figure 2h; and S5b, Supporting Information). It should be mentioned that the shifts of the N—H stretch and C=O stretch were not as conspicuous as the reported adduct interactions between the Lewis bases (the amino acids) and Pb^2+^.^[^ ^29^, ^43^, ^66^ ^]^ We postulate that this is because the lone pair electrons on N atom and O atom of the viruses already interact on their own due to the formation of the quaternary structure.

Transmission electron microscope (TEM) was used to observe perovskite precursor nucleation clusters. **Figure** **3**a shows that while the solution containing PbI_2_ and MAI forms circular perovskite crystal seeds, the 90 °C heated virus‐added solution forms the seeds around the M13 bacteriophage. High‐angle annular dark‐field imaging (HAADF) of scanning TEM (STEM) and the corresponding electron energy loss spectroscopy (EELS) analyses show the nucleation of MAPbI_3_ (Figure 3b). For the samples to which the M13 bacteriophage was added, the nucleation occurs along the network of the viruses (Figure 3c). Upon heating the sample at 90 °C, we could observe linear shafts of the viruses functioning as the nucleation site (Figure 3d). This confirms that the M13 bacteriophage can function as a nucleation site for MAPbI_3_ and affect the geometry of their crystal seeds.

**Figure 3:**
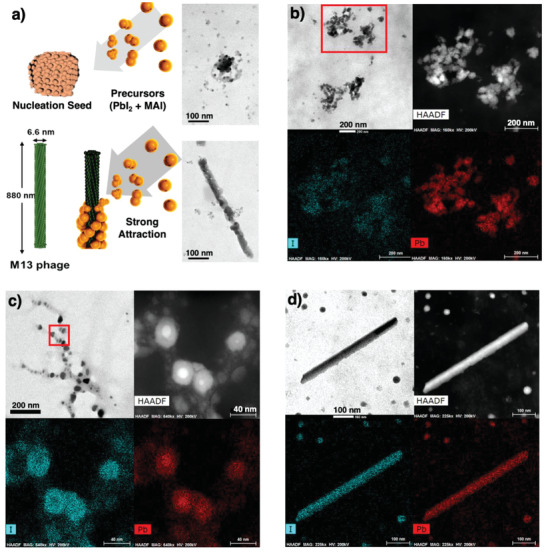
a) TEM images of the MAPbI_3_ solution (above) and the 90 °C heated virus‐added MAPbI_3_ solution (below). STEM image, HAADF image, and EELS analyses for I and Pb of b) the MAPbI_3_ solution, c) the r.t. virus‐added MAPbI_3_ solution, and d) the 90 °C heated virus‐added MAPbI_3_ solution.

Computational density functional theory (DFT) calculation was used to simulate the molecular interactions between the M13 bacteriophage and the perovskite materials. Interaction energy between the exposed four amino acids (A, K, D, and E) and the Pb^2+^ center shows that the negatively charged D and E interact much more strongly with Pb^2+^ than the neutral A and K, attributed to the strong coordination of the carboxylic anion to D and E. However, the interaction weakens when the perovskite precursor, PbI_2_ is introduced. This is because of the Coulombic repulsion between the carboxylic anion and the iodine atom of PbI_2_ (**Figure** **4**a; and Table S3, Supporting Information). In fact, the amino acid A shows a weaker interaction with both Pb^2+^ and PbI_2_. This is because both N atom and O atom in *α*‐amino acid structure (NH_2_CHCOOH) have low electron density due to the electron‐withdrawing inductive effect. This means that isolated amino groups and carboxylic groups should be considered when evaluating the interaction with the perovskite materials. K induces the strongest interaction with the perovskite crystal grain, then follows E, D, and A (Figure 4b; and Table S3, Supporting Information). This is because K has an isolated amino group far from the *α*‐amino acid. Amino groups have a smaller molecular size compared with carboxyl groups. Thus, the isolated amino group in K is more optimized for coordinating with the perovskite materials than the isolated carboxyl groups in D and E, which try to preserve the octahedron structure.^[^ ^67^ ^]^ The amino acid E, which has a negative charge on the carboxyl group induces slightly stronger interactions than the amino acid D. The inductive effect of the carboxyl group and the amino group in the amino acid D weakens the negative charge on the carboxyl end. TEM images of the perovskite film containing the M13 bacteriophage show an amorphous species with a thickness of ≈6.6 nm surrounding a perovskite grain (Figure 4d). This proves that a single strand of the viruses surround the perovskite grain as we predicted. We can expect the amino acids A, K, D, and E on the M13 bacteriophage to interact with the perovskite materials at the grain boundary.

**Figure 4:**
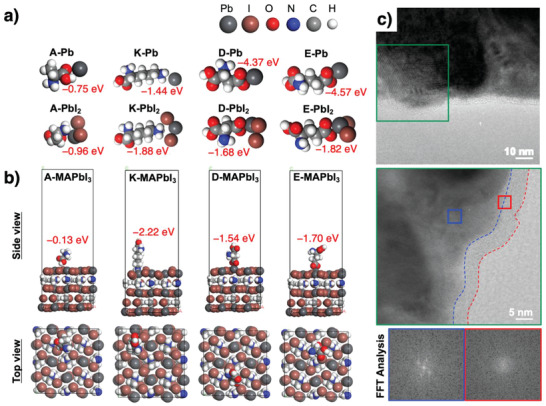
a) Computational modeling of the four types of amino acids from the M13 bacteriophage, a Pb atom, and PbI_2_. b) Computational modeling of an interaction between the four types of amino acids and the surface of a perovskite grain from the side view and the top view. c) TEM images of a string of M13 bacteriophage next to a perovskite grain along with their FFT analysis.

The crystal growth template effect of the virus on the entire perovskite film was examined using X‐ray diffraction spectroscopy (XRD). **Figure** **5**a shows that the perovskite film has a tetragonal phase with a dominant (110) peak at 14.1°.^[^ ^29^ ^]^ The intensity ratio of the (110) peak to the (220) peak indicates the growth of the (110)‐oriented grains.^[^ ^68^ ^]^ Addition of the M13 bacteriophage and heating the virus increased the (110)‐oriented grains, which is favorable for the hole transfer from the perovskite to the hole‐transporting layer (Table S4, Supporting Information).^[^ ^69^ ^]^ We conjecture this must have contributed to the enhancement of the FF in the denatured M13 virus‐added PSCs. Addition of the viruses increased the crystal grain size as evidenced by the decrease in the full‐width at half‐maximum (FWHM) value of the 110 peaks (Table S4 and Figure S6, Supporting Information).^[^ ^70^ ^]^ It is clear from the magnified and normalized (110) peaks in Figure 5b that the grain size increased significantly upon addition of the M13 bacteriophage. However, the increase in the grain size with heating the virus‐mixed perovskite solution is marginal. To confirm this, the SEM was used to observe the perovskite grain size with the heat treatments in different temperature from the top view and the cross‐sectional view. Compared with the reference perovskite film with no additives, the addition of the M13‐bacteriophage definitely increased the perovskite crystal size (Figure 5c; and Figures S7–S10, Supporting Information), confirming that the M13 bacteriophage functions as perovskite growth templates. When the virus‐added MAPbI_3_ precursor solution was treated at 65 °C before the film formation, the perovskite grains were slightly enlarged (Figure 5e). Further elevating the temperature to 90 °C increased the perovskite grains even more and the largest crystal grain in the image has a diameter of ≈640 nm (Figure 5g). The cross‐sectional SEM images depict a more conspicuous grain size enhancement (Figure 5d,f,h). While the grain size enhancement points to the increase in *J*_SC_ by the addition of the viruses and the denaturing of the viruses, the obtained increment in the *J*_SC_ from the devices is greater than what can be explained by the grain size enlargement alone.

**Figure 5:**
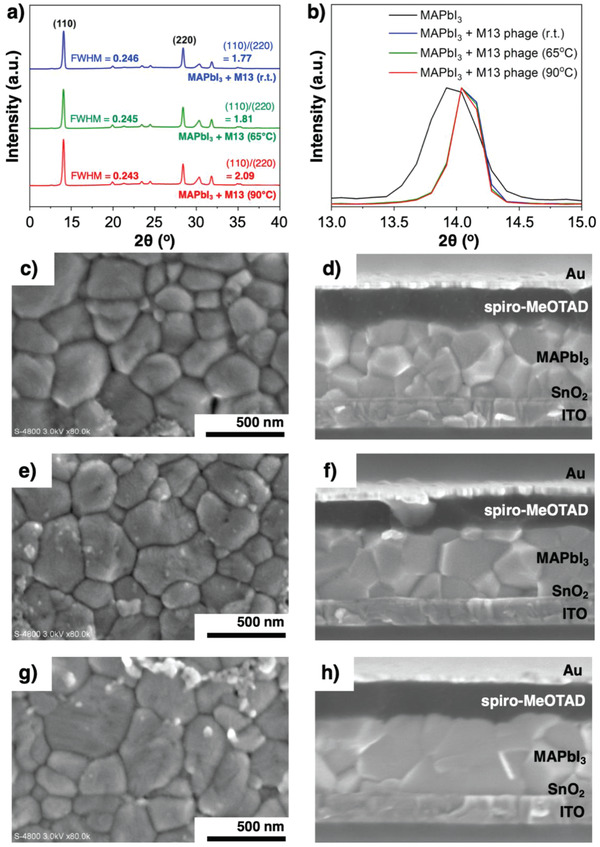
a) XRD spectra of MAPbI_3_ + M13 at room temperature (blue), MAPbI_3_ + M13 virus at 65 °C (green), MAPbI_3_ + M13 virus at 90 °C (red). b) (110) Peak showing the FWHM trend. The top‐view and cross‐sectional view SEM images of M13‐used perovskite films and M13‐used PSCs with different temperature treatment: c,d) for room temperature, e,f) for 65 °C, g,h) for 90 °C. (The top‐view SEM images contain remnants of spiro‐MeOTAD as the champion devices were used for the SEM analysis after washing spiro‐MeOTAD using CB).

In order to gain further insight into the increase in the *J*_SC_, UV–vis spectroscopy was conducted to characterize the light absorption of the virus‐added perovskite films (**Figure** **6**a). The virus‐added perovskite films show slightly extended absorption in the long‐wavelength region (Figure 6a inset). The absorption tail extends to the longer wavelengths with the increase in the perovskite precursor solution heating temperature. We ascribe this to the scattering effect of the added viruses and their morphology.^[^ ^10^, ^71^ ^]^ The calculated scattering spectra of the straight M13 bacteriophage and the bent M13 bacteriophage, emulating the viruses in r.t. and 90 °C, verify the phenomenon of the absorption tails (Figure 6b). This must have contributed to the *J*_SC_ increase as the external quantum efficiency (EQE) data also show the absorption tail in the long‐wavelength region in a similar fashion (Figure S11, Supporting Information).

**Figure 6:**
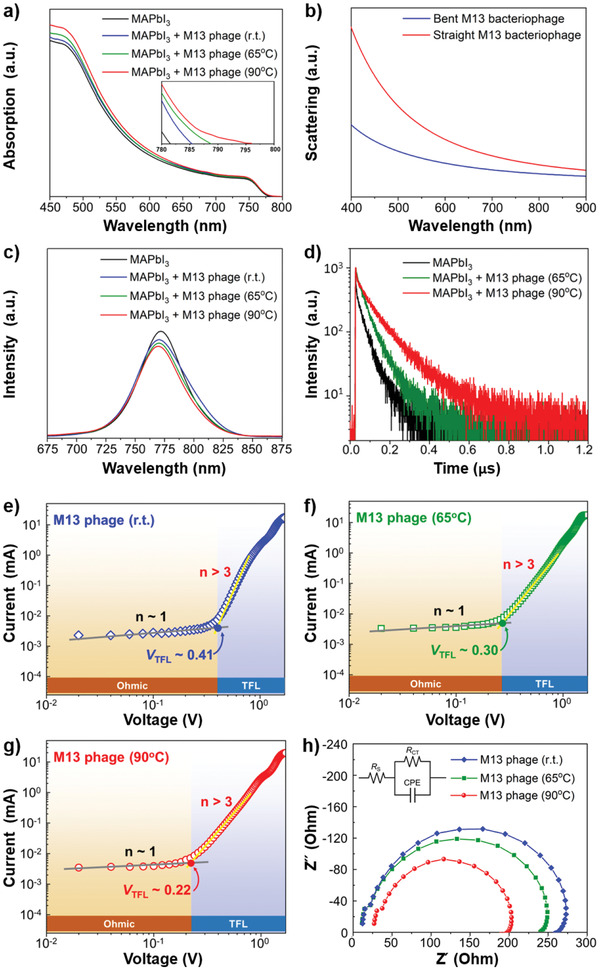
a) UV–vis spectra of glass/MAPbI_3_ (black), glass/M13 virus (r.t.) + MAPbI_3_ (blue), glass/M13 virus (65 °C) + MAPbI_3_ (green), glass/M13 virus (90 °C) + MAPbI_3_ (red). b) The simulated UV–vis spectra of scattering effect of bent M13 (blue) and straight M13 (red). c) Steady‐state PL spectra of ITO/MAPbI_3_ (black), ITO/M13 virus (r.t.) + MAPbI_3_ (blue), ITO/M13 virus (65 °C) + MAPbI_3_ (green), ITO/M13 virus (90 °C) + MAPbI_3_ (red). d) TRPL spectra of the reference MAPbI_3_ film (black), the M13 bacteriophage at 65 °C‐added MAPbI_3_ film (green), and the M13 bacteriophage at 90 °C‐added MAPbI_3_ film (red). The dark current–voltage curves of electron‐only devices e) ITO/SnO_2_/M13 virus (r.t.) + MAPbI_3_/PC_61_BM/Au (blue), f) ITO/SnO_2_/M13 virus (65 °C) + MAPbI_3_/PC_61_BM/Au (green), g) ITO/SnO_2_/M13 virus (90 °C) + MAPbI_3_/PC_61_BM/Au (red). h) Nyquist plots of the M13 virus (r.t.) + MAPbI_3_ (blue), M13 virus (65 °C) + MAPbI_3_ (green), M13 virus (90 °C) + MAPbI_3_ (red).

Steady‐state photoluminescence (PL) measurement was employed to investigate the passivation effect of M13 bacteriophage. The PL peaks of the virus‐added perovskite films on ITO substrates are significantly blueshifted compared with that of the reference perovskite film, verifying the passivation effect of the virus in perovskite films (Figure 6c).^[^ ^42^, ^52^, ^53^ ^]^ Moreover, FWHM of the PL peaks from the denatured virus‐added perovskite films is much narrower than that from the reference perovskite film, substantiating the reduction in the shallow trap density at the interface of the perovskite grains.^[^ ^23^, ^72^ ^]^ However, the PL peak of the virus‐added perovskite film in r.t. has a broader peak, which we suspect to be arising from the Urbach shallow defects states of undenatured viruses.^[^ ^73^ ^]^ Time‐resolved PL spectra (TRPL) of the reference perovskite film, the 65 °C heated virus‐added perovskite film and 90 °C heated virus‐added perovskite film on glass substrates were compared. Biexponential decay function fitter of the spectra shows that both of the samples possess similar decay time 1 (*τ*_1_) of ≈6 ns, which implies that the strength of passivation effect between the 65 °C heated virus and the 90 °C heated virus is similar (Figure 6d; and Table S5, Supporting Information). Yet, decay time 2 (*τ*_2_) was longer for the 90 °C heated virus‐added perovskite film than the 65 °C heated virus‐added perovskite film, indicating better film quality of the 90 °C heated virus‐added perovskite bulk which is attributed to the larger and more uniform perovskite grains. The average decay time (*τ*_ave_) was longer for the 90 °C heated virus‐added perovskite film, which explains the higher FF of the 90 °C heated virus‐added PSCs (Table 1). Electron trap density (*n*_t_) of the virus‐added perovskite films was calculated using the space‐charge‐limited current (SCLC) measurement by fabricating electron‐only devices (Figure 6e–g; and Figure S12, Supporting Information). Low trap‐filled limit voltage (*V*_TFL_) values in the virus‐added perovskite films fabricated from the elevated temperatures suggest reduced electron trap sites as *n*_t_ is proportional to *V*_TFL_. Table S6 (Supporting Information) shows the 90 °C heated virus‐added perovskite film has the lowest electron trap density of 7.78 × 10^15^ cm^−3^. Meanwhile, the 65 °C heated virus‐added perovskite film and r.t. virus‐added perovskite film exhibit *n*_t_ values of 1.04 × 10^16^ cm^−3^ and 1.35 × 10^16^ cm^−3^, respectively. The SCLC measurements reveal the passivation effect difference between the 90 °C heated viruses and the 65 °C heated viruses, which the TRPL data could not. Electrical impedance spectroscopy (EIS) was conducted to measure the charge transfer resistance (*R*_ct_) of the virus‐added perovskite films with different temperature treatments under illumination. The Nyquist plot was plotted along with an equivalent circuit depicted in the inset (Figure 6h). The numerical EIS data are summarized in Table S7 (Supporting Information). The 90 °C heated virus‐added perovskite sample showed the lowest *R*_ct_ of 166.4 Ω when the 65 °C heated virus‐added perovskite sample and r.t. virus‐added perovskite sample showed *R*_ct_ of 226.0 and 245.1 Ω, respectively. The lower *R*_ct_ implies reduced trap sites at the grain boundaries and efficiency charge flow. From this data, we can claim that the existence of the viruses themselves certainly do not hinder the charge flow. These data clarify the origins of the improved *V*_OC_ and FF of the denatured M13 bacteriophage‐added PSCs.

The addition of the M13 bacteriophage into the perovskite precursor led to the significant improvement in PCE of the PSCs. Denaturing the M13 virus at the right temperature (90 °C) increased the PCE even further by breaking down the inter‐ and intramolecular hydrogen bonding, which strengthened the interaction between the viruses and the perovskite materials as well as enhancing the light scattering effect. The improvement in *J*_SC_ was a result of both the grain size enlargement and the light absorption in the long‐wavelength region. The improvement in *V*_OC_ was evidenced by the improved charge transfer as a result of the reduced trap‐sites at the perovskite interface. Subsequently, the increase in FF followed. This work reveals that the biomaterials, such as the M13 bacteriophage, can be used in PSCs to improve their photovoltaic performance. As the previously reported additives for PSCs are chemically synthesized, use of environmentally harmful and carcinogenic organic solvents are inevitable, not to mention the high cost of mass production. The biologically cultured M13 bacteriophage is low‐cost, safe, and biodegradable. Therefore, our finding can bring a significant impact to the field of photovoltaics research as the first work incorporating biological materials in the system.

## Experimental Section

##### M13 Bacteriophage Synthesis

The M13 bacteriophage was purchased from New England Bio‐labs (Ipswich, MA). The product, which has a cat No. of M13KE (NEB, # N0316), was mass‐produced based on the following protocol; The M13 bacteriophage was amplified in an *Escherichia coli* (*E. coli*, ER2738) culture and purified by precipitating using polyethylene glycol, followed by dispersion. Then, it was refined through a membrane with 0.45 µm pores (Minisart NML, Syringe Filter 16 555) and centrifugated repeatedly. The accuracy of the cultured product was confirmed by DNA sequencing analysis (BIONICS, Seoul, Republic of Korea). The M13 bacteriophage concentration was measured using Nanodrop 2000 (Evolution 300, Thermo Fisher Scientific, Waltham, MA). Previously reported formulae and the absorbance spectral values were used for the concentration calculation.^[^ ^10^ ^]^

##### Perovskite Material Preparation

Unless otherwise noted, all materials including dry solvents were obtained from commercial suppliers, and used without additional purification. PbI_2_ (99.9985%) and methylammonium iodide (MAI) was purchased from Sigma‐Aldrich Inc. Anhydrous DMF, DMSO, and chlorobenzene (CB) were purchased from Alfa Aesar. Spiro‐MeOTAD was purchased from Luminescence Technology Corp. (Lumtec). SnO_2_ precursor solution was prepared by dissolving 6.8 mg SnCl_2_∙2H_2_O (Aldrich, > 99.995%) white powder in 1.0 mL of anhydrous ethanol (TCI), which was filtered by 0.45 µm polytetrafluoroethylene filter before using. For the preparation of CH_3_NH_3_PbI_3_ precursor solution, 461.0 mg of PbI_2_, 159 mg of CH_3_NH_3_I, DMSO, and virus contained DMSO solution (the volume of DMSO and virus solution was prepared in different ratio: a) virus‐free device: 71.0 µL DMSO; b) 0.01wt% virus‐used device: 51.0 µL DMSO and 20.0 µL of virus solution; c) 0.02 wt% virus‐used device: 31 µL DMSO and 40 µL virus solution; d) 0.03 wt% virus‐used device: 11 µL DMSO and 60 µL virus solution) were mixed in 635.0 µL of DMF solution at r.t. with stirring for 1 h. For denaturing virus, the thermal treatment was next conducted at 65 or 90 °C for 15 min, respectively. The perovskite solution was directly applied or filtered through a 0.45 µm polytetrafluoroethylene filter prior to use. The spiro‐MeOTAD solution was prepared by mixing 34.3 mg spiro‐MeOTAD, 7.7 µL of a stock solution of 520.0 mg mL^−1^ lithium bis(trifluoromethylsulphonyl)‐imide in anhydrous acetonitrile, and 13.5 µL of 4‐*tert*‐butylpyridine in 1.0 mL anhydrous chlorobenzene.

##### Device Fabrication

ITO substrates, prepatterned ITO/glass substrates (12 ohm sq.^−1^, 15 × 15 mm^2^) (AMG Tech) were cleaned and sonication with detergent, distilled water, acetone and isopropanol in an ultrasonic bath for 15 min, respectively. Next, the cleaned ITO substrates were treated with UV/O_3_ for 15 min. Subsequently, 25.0 µL of SnO_2_ precursor solution was spin‐coated on the cleaned ITO substrate at 3000 rpm for 30 s, which was annealed at 125 °C for 5 min and followed by annealing at 150 °C for 30 min. After cooling down to r.t., the spin‐coating process was repeated one more time followed by annealing at 150 °C for 5 min and then 190 °C for 1 h. After that, the SnO_2_ coated ITO glass was further treated with UV/O_3_ for 15 min. Next, 25.0 µL of perovskite precursor solution was spin‐coated on the SnO_2_ layer at 4000 rpm for 20 s, with slowly dropping 0.3 mL of anhydrous diethyl ether onto the substrate 5 s after the start of the spin‐coating process, followed up with annealing at 65 °C for 1 min and 100 °C for 5 min. The hole transporting layer was spin‐coated from the 20.0 µL of spiro‐MeOTAD solution at 4000 rpm for 20 s. Finally, a 60 nm thick of Au anode was fabricated by thermal deposition at a constant evaporation rate of 0.05 nm s^−1^ under pressure of 10^−6 ^torr. inside a thermal evaporator.

##### Device Characterizations

*J–V* curves of perovskite solar cells under light were measured using a source meter (Keithley 2400, Tektronix) under the simulated sunlight irradiation of 1 sun (AM 1.5G; 100 mW cm^−2^) using a solar simulator (EMS‐35AAA, Ushio Spax Inc.) with an Ushio Xe short arc lamp 500. The source meter was calibrated using a silicon diode (BS‐520BK, Bunkokeiki). When evaluating, devices were masked with a black aperture to set the active area of the device to 0.1 cm^2^. The scan directions of forward (from −0.2 to 1.2 V) and reverse (from 1.2 to −0.2 V) were used. The forward scan was measured followed by a reverse scan. A scan rate of 100 mV s^−1^ with a 0.02 step and 100 ms waiting time was used for the measurement. SEM measurements were carried out on an S‐4800 scanning electron microscopy (Hitachi). Shimadzu IRAffinity‐1s was used for the FTIR measurement. Steady‐state PL spectra were measured using a PL spectrometer (JASCO Spectrofluorometer FP‐8300) with Xenon as the excitation source (excitation at 560 nm). The PL spectra were analyzed by a Horiba Jobin Yvon system, where a 640 nm monochromatic laser was used as an excitation source. TRPL spectra were obtained using a Picoharp 300 with time‐correlated single‐photon counting capabilities. Perovskite films on glass were excited by a 640 nm pulse laser with a repetition frequency of 200 kHz provided by a picosecond laser diode head (PLD 800B, PicoQuant). EQE spectra were measured using machine spectrometer with a wavelength ranging from 300 to 850 nm. For the computational study, generalized gradient approximation (GGA)‐level spin‐polarized DFT calculation was performed using the generalized gradient approximation with the Perdew–Burke–Ernzerhof^[^ ^74^ ^]^ exchange‐correlation functional. All DFT calculations were performed using the Vienna ab initio simulation package (VASP). The cut‐off energy of plane‐wave expansions was set to 400 eV. DFT‐D2 method of Grimme^[^ ^75^ ^]^ was employed to treat the van der Waals terms. The convergence criteria for solving electronic wave function and local minima were 1 × 10^−5 ^eV. All geometries were converged to within 3 × 10^−2 ^eV Å^−1^ for maximal forces. STEM and EELS were conducted using H‐7600 (Hitachi) and 200 kV field emission transmission electron microscope (TALOS F200X) with fast analytical energy‐dispersive X‐ray spectroscopy. *n*_t_ and *V*_TFL_ were measured using SCLC in electron‐only devices (ITO/SnO_2_ (30 nm)/MAPbI_3_ (with or without virus added) (400 nm)/PC_61_BM (30 nm)/Au (60 nm)). The *n*_t_ and *V*_TFL_ values are calculated from the equation, *V*_TFL_ = *n_t_ed* ^2^/2*ε*_0_ *ε*_r_, where *e* is electric charge (1.602 × 10^−19 ^V m^−1^), *d* is the thickness of the active layer, *ε*_0_ is the vacuum permittivity (8.85 × 10^−14^ F cm^−1^), and *ε*_r_ is the relative dielectric constant taken as 46.9 *V*_TFL_ = *n_t_ed* ^2^/2*ε*_0_ *ε*_r_, where *e* is electric charge (1.602 × 10^−19 ^V m^−1^), *d* is the thickness of the active layer, *ε*_0_ is the vacuum permittivity (8.85 × 10^−14^ F cm^−1^), and *ε*_r_ is the relative dielectric constant taken as 46.9.^[^ ^76^ ^]^ The experimental dark current density was measured under an applied voltage swept from 0 to −3 V. EIS measurements were conducted by Solartron Analytical 1255B under AM1.5G light condition.

## Conflict of Interest

The authors declare no conflict of interest.

## Supporting information

Supporting Information
