# Supplementary material for: Denatured M13 Bacteriophage‐Templated Perovskite Solar Cells Exhibiting High Efficiency
Source: Adv Sci (Weinh). 2020 Aug 5;7(20):2000782. doi: 10.1002/advs.202000782 (PMC7578877; doi:10.1002/advs.202000782)
Supplement: Supplementary file 1 — Supporting Information [file ADVS-7-2000782-s001.pdf]

2020 Wiley-VCH GmbH

ADVANCED SCIENCE
Open Access

Supporting Information

for *Adv. Sci.,* DOI: 10.1002/advs.202000782

Denatured M13 Bacteriophage-Templated Perovskite Solar Cells Exhibiting High Efficiency

Hao-Sheng Lin, Jong-Min Lee, Jiye Han, Changsoo Lee, Seungju Seo, Shaun Tan, Hyuck Mo Lee, Eun Jung Choi, Michael S. Strano, Yang Yang, Shigeo Maruyama, Il Jeon, Yutaka Matsuo,* and Jin-Woo Oh**

Copyright WILEY-VCH Verlag GmbH & Co. KGaA, 69469 Weinheim, Germany, 2020.

Supporting Information

Denatured M13 Bacteriophage-Templated Perovskite Solar Cells Exhibiting High Efficiency

Hao-Sheng Lin, Jong-Min Lee, Jiye Han, Changsoo Lee, Seungju Seo, Shaun Tan, Hyuck Mo Lee, Eun Jung Choi, Michael S. Strano, Yang Yang, Shigeo Maruyama, Il Jeon, Yutaka Matsuo*, and Jin-Woo Oh**

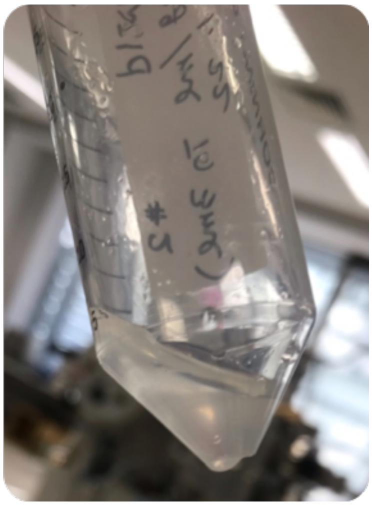

Figure S1. Picture of a M13 bacteriophage suspension with a concentration of 6.2 mg mL^{-1} in anhydrous DMSO.

Table S1. Prepared amounts of the M13 bacteriophage in the perovskite precursor solutions

Entry	Precursor	DMSO		M13 phage solution		wt% of phage	wt% of $MAPbI_3$	wt% of DMSO
		mass	volume	mass	volume			
Ref. (0%)	200.0 mg	12.5 mg	11.4 µL	0.000 mg	0.0 µL	0.0000%	94.0999%	5.9001%
0.03%	200.0 mg	0.0 mg	0.0 µL	0.071 mg	11.4 µL	0.0333%	94.0999%	5.9001%
0.02%	200.0 mg	5.1 mg	4.6 µL	0.042 mg	6.8 µL	0.0198%	94.0999%	5.9001%
0.01%	200.0 mg	8.8 mg	8.0 µL	0.021 mg	3.4 µL	0.0099%	94.0999%	5.9001%

Table S2. Photovoltaic parameters of the PSCs with different concentrations of the M13 bacteriophage added under 1 sun (AM 1.5 G, 100 mW cm^{-2}). Average values with standard deviations were obtained from the devices of the same batch.[a)]

Entry	concentration (wt%)	J_{SC} (mA cm^{-2})	V_{OC} (V)	FF	PCE_{best}	$PCE_{average}$
1	0	23.6 ± 0.1	1.035 ± 0.008	0.700 ± 0.009	17.8%	17.1 ± 0.4%
2	0.01	23.6 ± 0.1	1.048 ± 0.010	0.700 ± 0.005	18.2%	17.7 ± 0.4%
3	0.02	24.4 ± 0.2	1.058 ± 0.004	0.706 ± 0.009	18.7%	18.4 ± 0.2%
4[b)]	0.02	24.3 ± 0.6	1.057 ± 0.004	0.693 ± 0.020	18.3%	17.8 ± 0.2%
5	0.03	23.9 ± 0.2	1.047 ± 0.010	0.700 ± 0.020	18.1%	17.5 ± 0.5%

[a)] Unless otherwise specified, all perovskite precursor solutions were filtered through a 0.45-μm-polytetrafluoroethylene-filter without heat treatment. [b)] The perovskite precursor solution was used without filtration.

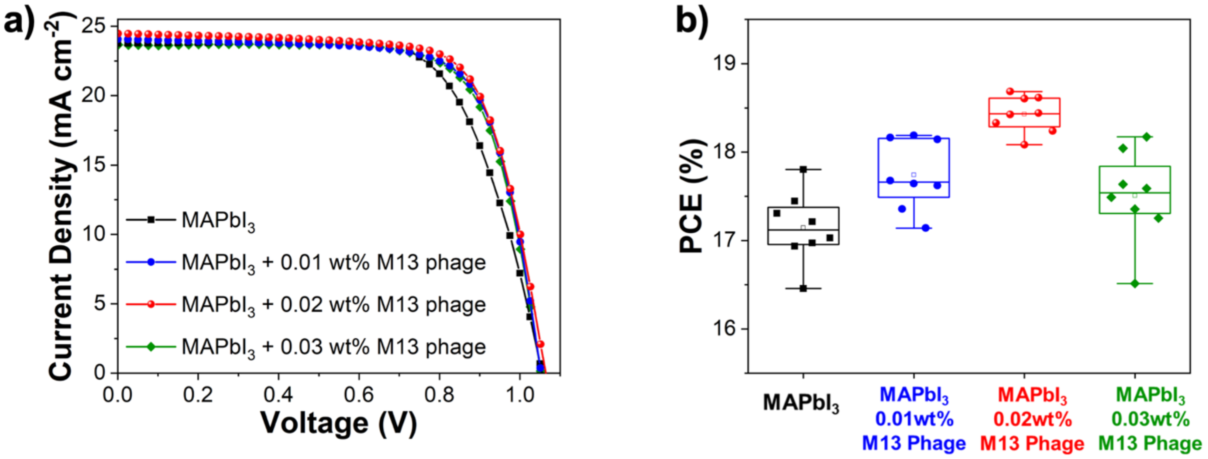

Figure S2. a) *J*–*V* curves of the best-performing M13 bacteriophage-added PSCs with different virus concentrations. b) The box plot chart showing the PCEs of the M13 bacteriophage-added PSCs with different M13 bacteriophage concentrations.

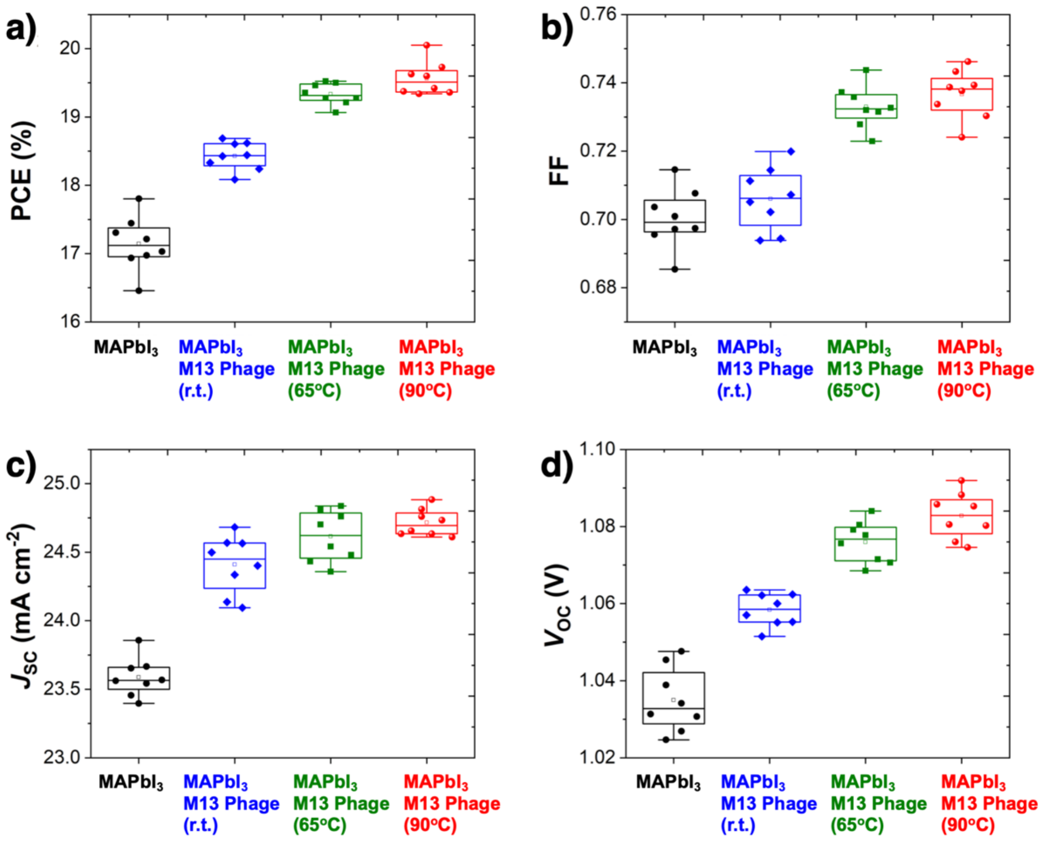

Figure S3. Statistical analyses of the photovoltaic parameters of PSCs using 0.02 wt% M13 bacteriophage with different temperature treatments.

Table S3. Intermolecular interaction energies between the amino acids and the perovskite materials derived from the DFT calculation using VASP.

Molecules	A-Pb	K-Pb	D-Pb	E-Pb
Interaction Energy	-0.75 eV	-1.44 eV	-4.37 eV	-4.57 eV
Molecules	A-PbI_2	K-PbI_2	D-PbI_2	E-PbI_2
Interaction Energy	-0.96 eV	-1.88 eV	-1.68 eV	-1.82 eV
Molecules	A-perovskite	K-perovskite	D-perovskite	E-perovskite
Interaction Energy	-0.13 eV	-2.22 eV	-1.54 eV	-1.70 eV

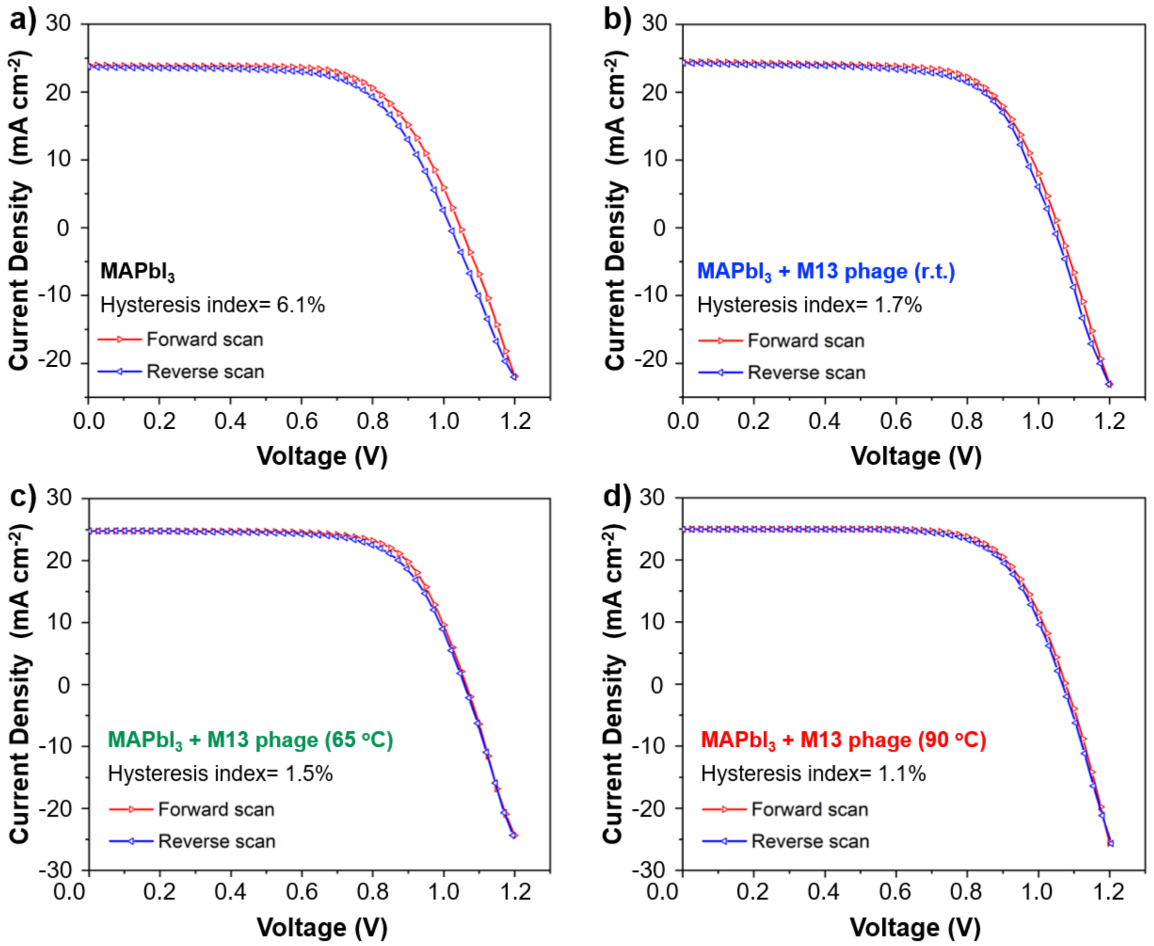

Figure S4. *J–V* curves of forward scan (blue) and reverse scan (red) for a) the reference device, b) the M13 virus (r.t.)-added device, c) the M13 virus (65 °C)-added device, and d) the M13 virus (90 °C)-added device with the hysteresis indices indicated for every case.

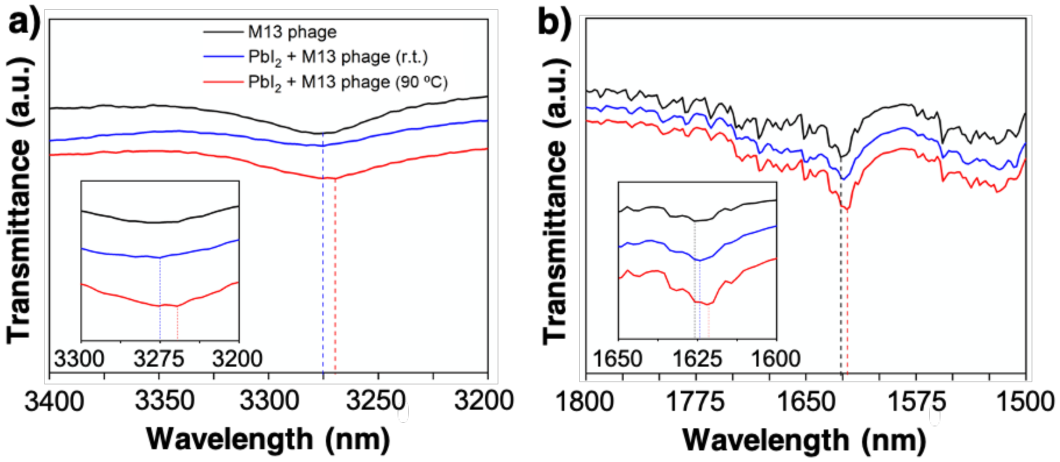

Figure S5. FTIR spectra of a) $\nu_{(N-H)}$ and b) $\nu_{(C=O)}$ the M13 bacteriophage (black), the adduct powder of PbI_2 mixed with the M13 bacteriophage (blue) and the heat-treated adduct powder (red) in solution state with magnified insets showing the peak shifts clearly (Figure 2e and 2h).

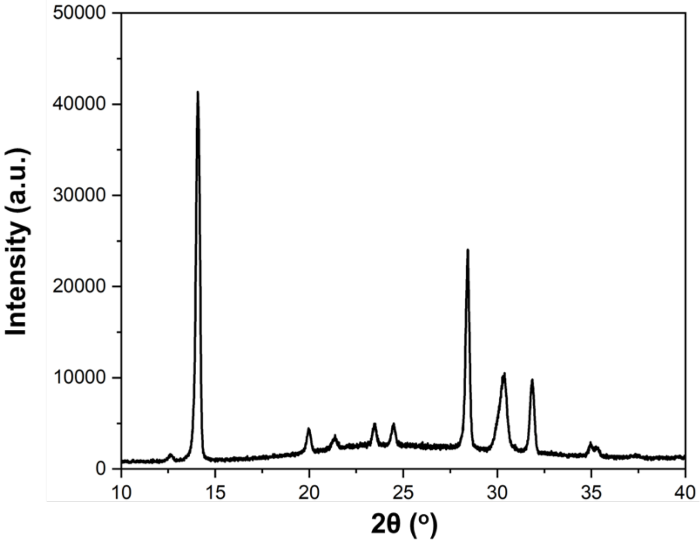

Figure S6. XRD spectrum of reference $MAPbI_3$.

Table S4. XRD peak characterization and information.

Sample	Peak	Position (°)	Intensity	Peak Area	FWHM	Peak Ratio
ITO/$MAPbI_3$	<110>	14.1	41344.34	10966.47	0.249	1.72
	<220>	28.4	24062.72	5921.62	0.260	
ITO/$MAPbI_3$+M13 phage (r.t.)	<110>	14.1	47773.73	12879.08	0.246	1.77
	<220>	28.4	27045.01	6952.35	0.249	
ITO/$MAPbI_3$+M13 phage (65°C)	<110>	14.1	39639.55	10633.00	0.245	1.81
	<220>	28.4	21937.49	5328.94	0.247	
ITO/$MAPbI_3$+M13 phage (90°C)	<110>	14.1	46601.38	12576.87	0.243	2.09
	<220>	28.4	22349.56	5453.45	0.249	

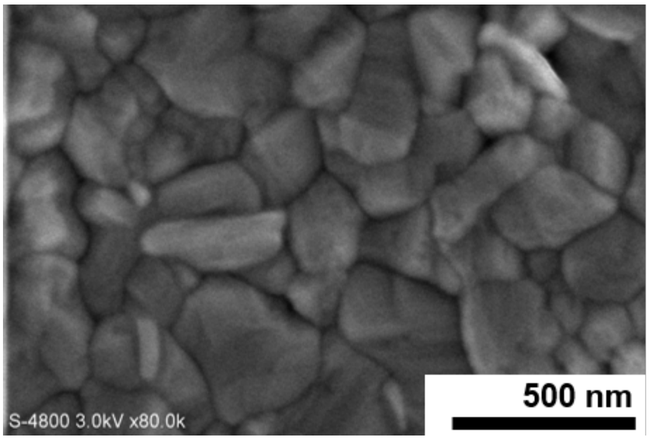

Figure S7. Top-view SEM image of the reference perovskite film (no additives).

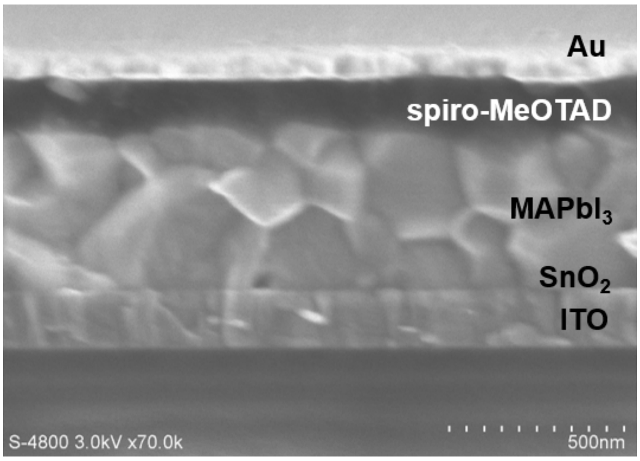

Figure S8. Cross-sectional view SEM image of reference PSC.

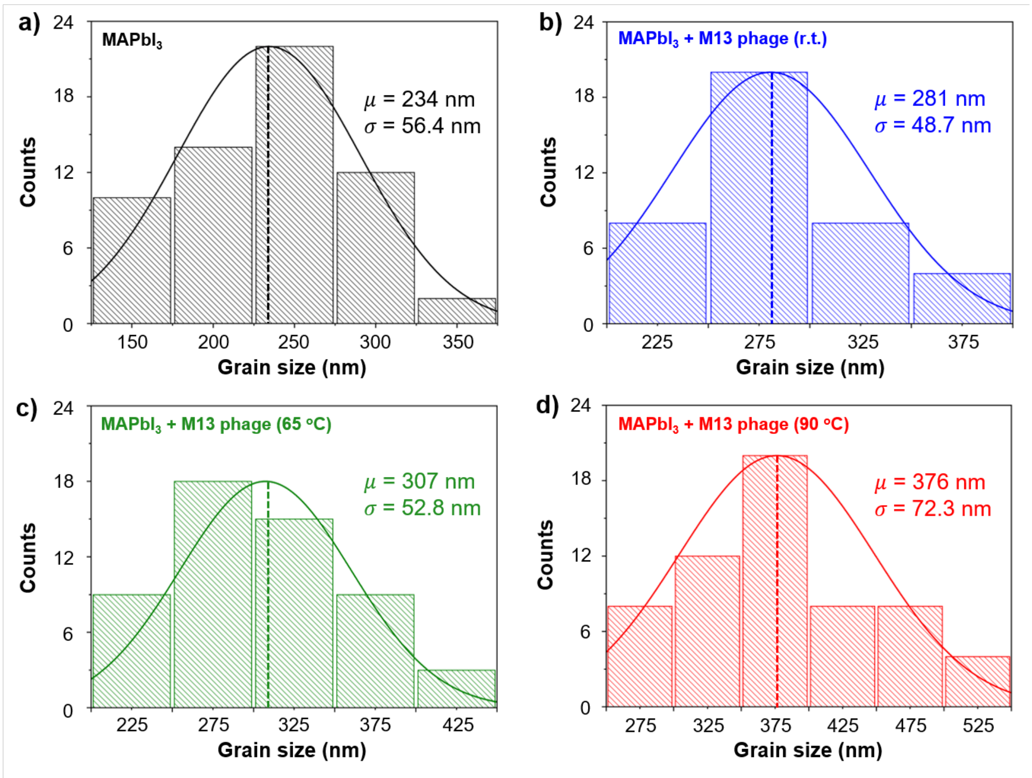

Figure S9. The statistical distribution of the perovskite grains with the mean value, μ and the standard deviation, σ for a) the reference $MAPbI_3$ film, b) the M13 virus (r.t.)-added $MAPbI_3$ film, c) M13 virus (65 °C)-added $MAPbI_3$ film, and d) M13 virus (90 °C)-added $MAPbI_3$ film.

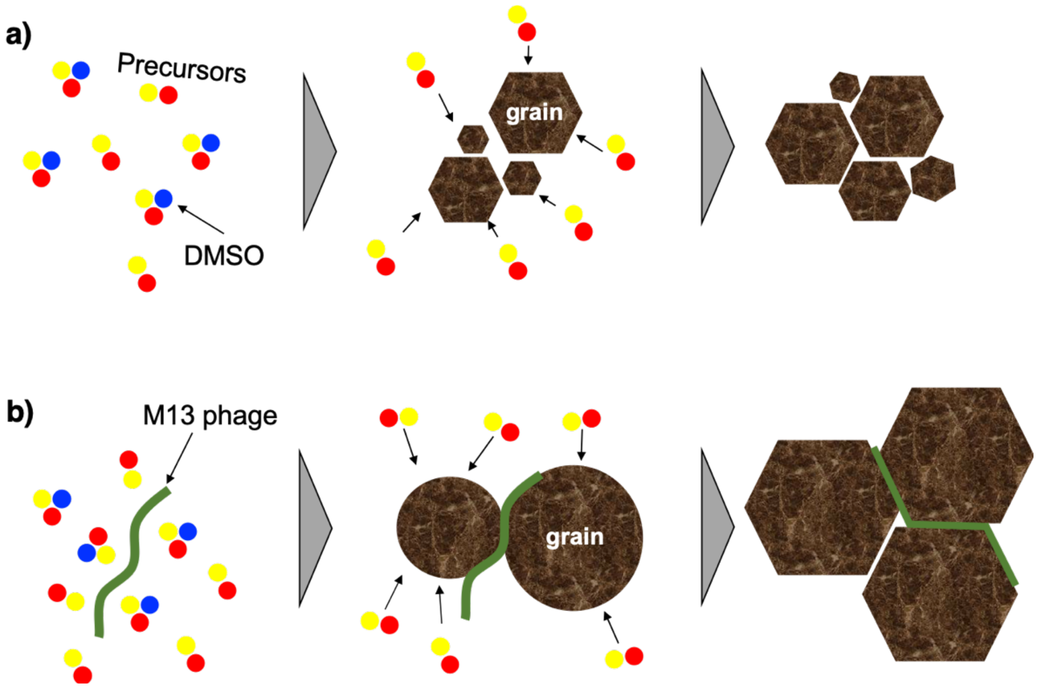

Figure S10. Illustration of perovskite crystal growth mechanism a) without the M13 phage and b) with the M13 phage.

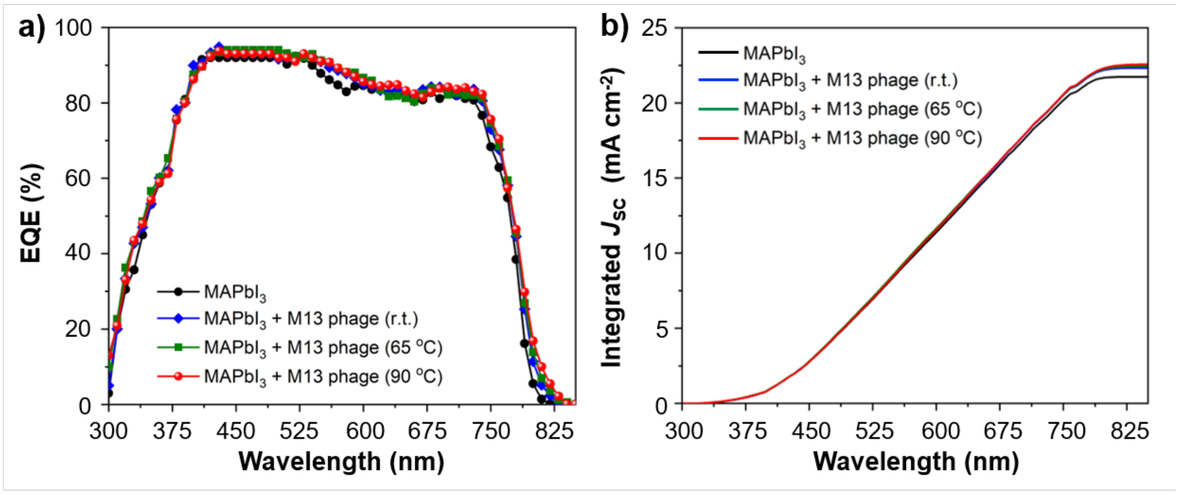

Figure S11. a) EQE data and b) J_{SC} values from the integrated EQE of the PSCs fabricated in this work.

Table S5. The fitting parameters for time-resolved photoluminescence spectra.

Device	A_1	Proportion (%)	τ_1 (ns)	A_2	Proportion (%)	τ_2 (ns)	τ_{ave} (ns)
$MAPbI_3$	651.4	54.5	7.1	544.2	45.6	56.2	30.3
$MAPbI_3$ + M13 phage (65 °C)	290.4	27.2	6.4	778.0	72.8	50.0	38.5
$MAPbI_3$ + M13 phage (90 °C)	481.9	43.4	6.1	628.6	56.5	91.8	54.6

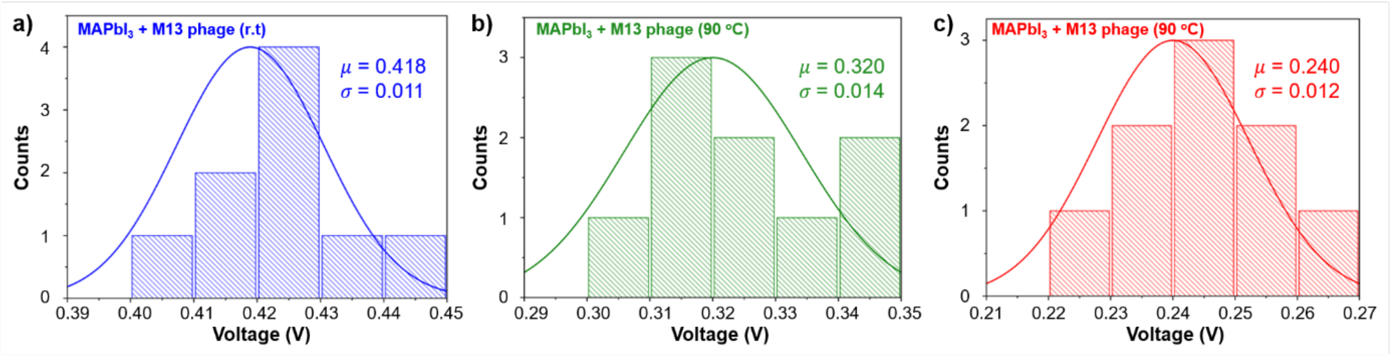

Figure S12. The statistical distribution curves with μ as mean value, σ as standard deviation, for evaluating the trap density of a) the M13 virus (r.t.)-added PSCs, b) the M13 virus (65 °C)-added PSCs, and c) the M13 virus (90 °C)-added PSCs.

Table S6. Average n_t values of the virus-added perovskite films with different temperatures.

Temperature (°C)	V_{TFL} (V)	n_t (cm^{-3})
r.t.	0.418 ± 0.022	(1.35 ± 0.08) × 10^{16}
65	0.320 ± 0.014	(1.04 ± 0.05) × 10^{16}
90	0.240 ± 0.024	(7.78 ± 0.08) × 10^{15}

Table S7. Numerical EIS fitting data of the M13 virus-added perovskite films with different temperatures..

Temperature (°C)	R_S (Ω)	R_{ct} (Ω)
r.t.	131.5 ± 2.0	245.1 ± 1.8
65	119.8 ± 2.1	226.0 ± 1.2
90	94.3 ± 1.3	166.4 ± 2.2
